# Intravascular lithotripsy: A novel option for severe calcification of coronary artery

**DOI:** 10.1002/clc.24186

**Published:** 2023-11-09

**Authors:** He Lv, Xinyu Li, Zengduoji Ren, Xuelian Ma, Zhilu Qin, Qiang Fu

**Affiliations:** ^1^ Department of Cardiology The People's Hospital of China Medical University Shenyang City Liaoning Province People's Republic of China; ^2^ Department of Cardiology Jinzhou Medical University Jinzhou City Liaoning Province People's Republic of China; ^3^ Department of Cardiology, The Fourth Affiliated Hospital of China Medical University The People's Hospital of China Medical University Shenyang City Liaoning Province People's Republic of China

**Keywords:** intravascular lithotripsy, shockwave ballon, severe calcification, precautions and coping strategies, treatment algorithm

## Abstract

**Background:**

Severe coronary artery calcification is associated with low success rate of interventional operation, perioperative adverse cardiac events, and poor prognosis, which is a major problem faced by operators. The existing therapy methods all have inherent limitations, such as unsatisfactory balloon crossability, inadequate balloon dilation, and so on. The emergence of intravascular lithotripsy (IVL) has brought the dawn of the treatment of calcified lesions by using unfocused acoustic pressure waves to fracture calcification in situ. And IVL is the only technology capable of targeting deep calcification.

**Hypothesis:**

IVL may have great clinical application values and potential prospects.

**Method:**

Based on the existing clinical evidence of IVL and traditional treatment ways, this review discusses the safety and efficacy of IVL. Combined with clinical practice, the precautions and coping strategies of IVL are analyzed. And the review improves the management algorithm of coronary calcification.

**Results:**

IVL has extremely high safety and effectiveness for severe coronary calcification compared with other ways, and structural improvements of IVL will further expand its value.

**Conclusions:**

The emergence of IVL could set off a revolution in the treatment of coronary artery calcification.

## Graphical abstract

An algorithm for coronary calcification. CAG, coronary angiography; CaLADeN, calcium length, arc, vessel diameter, and nodule by IVUS; CVI, calcium volume index from OCT; IVL, intravascular lithotripsy; IVUS, intravascular ultrasound; NC, Noncompliant; NSE, nonslip element; OA, orbital atherectomy; OCT, optical coherence tomography; OPN, ultrahigh pressure noncompliant balloon; RA, rotational atherectomy.

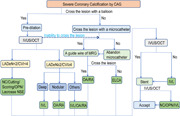

**Abbreviations**
CAG: coronary angiography
ELCA: excimer laser coronary atherectomy
IVL: intravascular lithotripsy
IVUS: intravascular ultrasound
MACE: major adverse cardiac events
MI: myocardial infarction
OCT: optical coherence tomography
RA: rotational atherectomy

## 1 INTRODUCTION

Coronary calcification is closely related to age, atherosclerosis, diabetes, and chronic kidney disease,^1^ so calcification is not uncommon in coronary artery. Coronary stenosis with severe calcification implies decreased vascular compliance and abnormal vasomotor response, which leads to further diminished myocardial perfusion. Studies have shown that the severity degree of calcification affects the survival rate of patients, the incidence of myocardial infarction (MI), and target vascular revascularization.^2^ To date, coronary calcification is still an obstacle to further improvement of percutaneous interventional therapy, and it often predicts a poor clinical prognosis. This dilemma has facilitated the development of many therapeutic approaches, such as cutting balloon, rotational atherectomy (RA), excimer laser coronary atherectomy (ELCA), and so on. However, they all have inherent limitations, such as unsatisfactory balloon crossability, inadequate balloon dilation, or slightly high rate of complications.^3^ Intravascular lithotripsy (IVL), a novel technology derived from sound waves in the treatment of kidney stones, brings the dawn of the treatment of calcified lesions. IVL uses acoustic pressure waves to crack calcification in situ, and is the only technique that can work on deep calcification.^1^ Based on the existing clinical evidence of IVL and traditional treatment ways, this review discusses the safety and efficacy of IVL. The precautions and coping strategies of IVL are analyzed combined with clinical practice. Besides, the review improves the management algorithm of coronary calcification.

## 2 INVASIVE IMAGING DIAGNOSIS METHODS

Computed tomography coronary angiography (CAG) is a noninvasive way to detect vascular calcification, but it provides finite value during the process of interventional therapy. In practice, the evaluation of calcified lesions relies more on CAG, intravascular ultrasound (IVUS), or optical coherence tomography (OCT).^4^

### 2.1 CAG

CAG is the only way to start interventional therapy and is also a means to preliminarily evaluate coronary calcification. CAG can detect 38% calcification lesions,^5^ which can be divided into four parts: (1) There is no calcification; (2) mild calcifications mean light and fuzzy high‐density shadows can be seen only when a heart beats, and the heart does not beat without the presence of calcification shadow; (3) a moderate calcification is a evident and easy to see high‐density shadow when the heart beats; (4) severe calcifications mean clear high‐density shadows both with and without beating heart. Calcification in CAG is manifested as high‐density shadow with uneven density along vessels. Therefore, CAG, this nonendovascular imaging method, has high specificity for the diagnostic performance of calcification lesions. Conversely, the sensitivity of CAG is only 48%.^6^

### 2.2 IVUS

Calcification in IVUS appears as a bright and white image of the lesion surface followed by a black sound shadow, often accompanied by multiple reflections. The sensitivity and specificity of IVUS in the diagnosis of calcified lesions are higher than CAG.^6^ According to the location distribution of calcification, calcified plaques can be divided into superficial calcification (located in the inner membrane and in contact with the lumen), deep calcification (near the junction of the media and outer membrane), and mixed calcification.^7^ The IVUS calcification score (Figure S1) indicated that both lesions with a calcification angle >270° and a length >5 mm, with a 360° annular calcification, with a reference vessel diameter ≤3.5 mm and with calcification nodules were given one point, respectively.^8^ IVUS can provide sufficient spatial resolution, penetration depth, and real‐time vascular cross‐sectional images,^9^ but ultrasonic waves are reflected by deposited calcium, accompanied by black sound shadows behind calcification. Hence IVUS cannot evaluate the range and depth of calcification.^1^

### 2.3 OCT

Calcification lesions in OCT often appear as areas of low or uneven signal with sharp edges. OCT can automatically quantify the thickness, area, volume, and other parameters of calcification. Based on the above parameters, calcification lesions are classified into the following four types: (1) An annular calcification means that the angle of calcified plaque is >270°; (2) a punctate calcification refers to the angle ≤90° and length <10 mm; (3) a deep calcification means that the distance of the calcified plaque from the lumen is >100 μm; (4) a superficial calcification means that the calcified plaque is 65–100 μm away from the lumen.^6^ An OCT scoring system (Figue S1) was proposed to evaluate the severity of calcification lesions: There are two points for calcification angle >180° and one point for calcification angle 90–180°. The calcification thickness >0.5 mm is one point, and the calcification length >5 mm is one point.^8^ OCT has a limited penetration capacity, but light can penetrate calcification without being reflected.^1^ Therefore, calcified plaques have low back reflection and low attenuation in OCT images. OCT is more sensitive than IVUS in the diagnosis of calcified lesions but has slightly less specificity than IVUS.^6^

## 3 CORONARY INTRAVASCULAR LITHOTRIPSY SYSTEM

### 3.1 System composition

**Figure 1:**
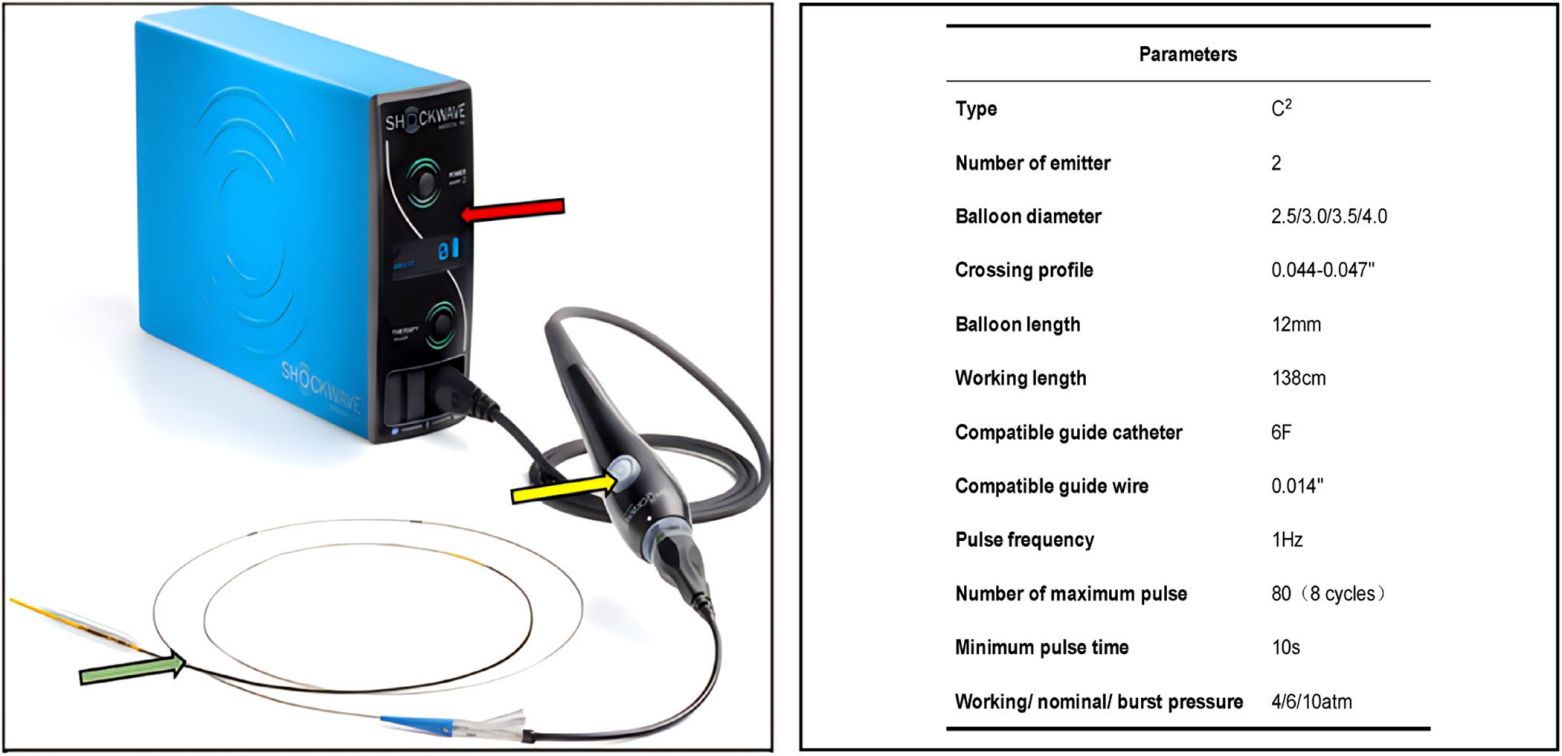
The structure and parameters of intravascular lithotripsy. The red arrow points to pulse generator. The yellow arrow points to connector cable. The blue arrow points to C2‐type shockwave balloon catheter. Adapted from Neleman et al.^10^ and Kereiakes et al.^11^

IVL (Shockwave Medical Inc.) system consists of two parts: intravascular shockwave therapy apparatus and C2‐type shockwave balloon catheter. Balloon catheter (working length: 138 cm) is a semi‐compliant rapid exchange balloon (working/nominal/burst pressure: 4/6/10 atm) with two pulse emitters, and the length is 12 mm. The outer diameter of the balloon ranges from 0.044 to 0.047″, and the diameter ranges from 2.5 to 4.0 mm (the increment is 0.5 mm). The balloon contains a mixture of saline and contrast agent. C2‐type catheter allows the use of 0.014″ guide wire, and it can be compatible with 6 F guide catheter and guide extension catheter. The magnetic connector cable and the portable rechargeable pulse generator (size: 28 cm × 15.2 cm × 29.2 cm, weight: 6.8 kg) constitute the intravascular shockwave therapy apparatus (Figure 1).^1^, ^10^

### 3.2 Working principle

**Figure 2:**
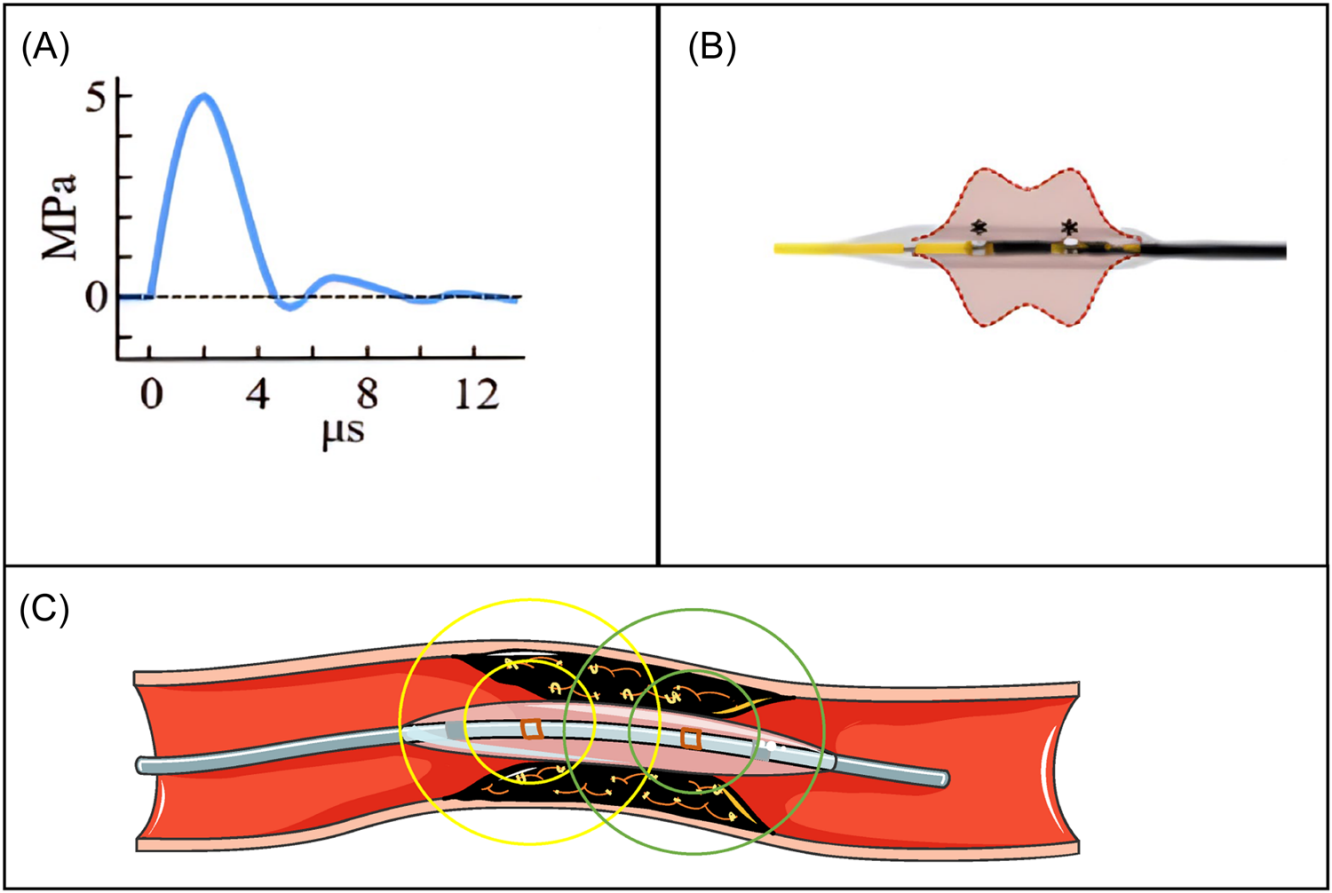
The acoustic pressure waves of intravascular lithotripsy. (A) Waveform of unfocused acoustic pressure waves. (B) Activation energy diagram of the central channel between the two emitters. (C) Propagation diagram of acoustic pressure waves. Adapted from Kereiakes et al.^11^

The IVL system emits about 3000 V of electric energy through the IVL generator, which is transmitted to the pulse emitter in the balloon to generate electrical sparks. The above process can make the liquid in the balloon produce rapidly expanding and collapsing steam bubbles. Thus, unfocused acoustic pressure waves of about 50 atm are formed, which realizes the conversion of electrical energy to mechanical energy. The unfocused waves are associated with low energy flux density, that is, low degree of tissue damage, and the peak negative pressure generated by acoustic pressure waves can be negligible. The resulting tensile stress (about 0.3 MPa or 3 atm) is well below the threshold of tissue damage, thereby mitigating the cavity‐induced tissue damage. The activation energy of the central channel between the two emitters is shared (Figure 2). When the acoustic pressure wave encounters tissues with impedance differences or acoustic mismatches, it will produce a local detonation phenomenon. The solid material will be cracked, and waves can continue to propagate and gradually decay in the liquid or liquid‐like soft tissue. The two electrodes of IVL are enclosed in a liquid‐filled balloon, which protects the electrodes and dissipates the heat generated during the formation of steam bubbles for reducing thermal damage to the tissue. The acoustic pressure wave penetrates to a depth of 3–7 mm, selectively destroying both superficial and deep calcification. IVL minimizes the damage of the surrounding tissue and changes the vascular compliance with the C2‐type IVL catheter balloon.^1^, ^11^, ^12^

### 3.3 Operation process

**Figure 3:**
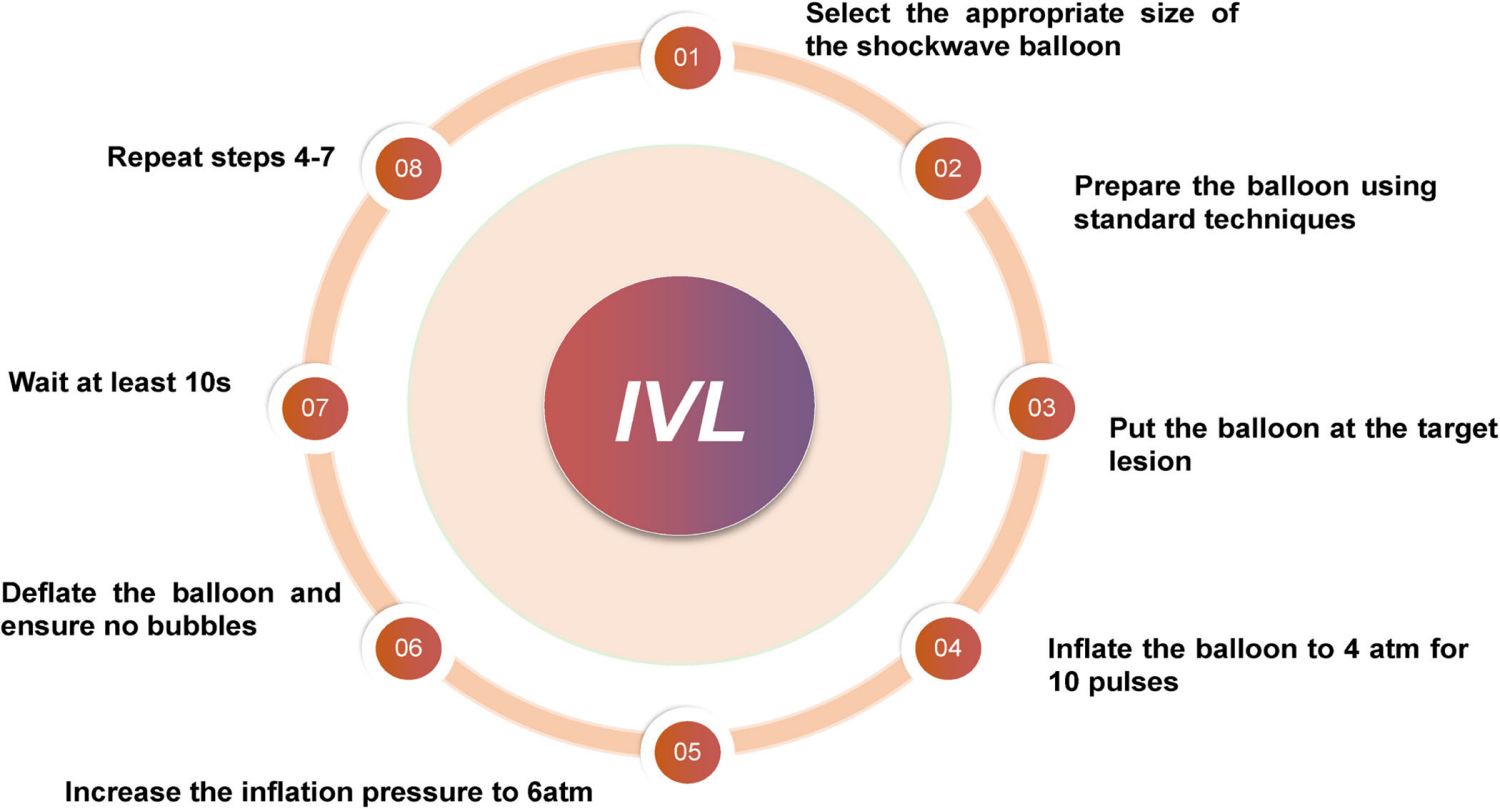
The operation flow of intravascular lithotripsy. Adapted from Neleman et al.^10^

The operation process of IVL is not complicated, the main steps are as follows: (1) Select the appropriate size of the shockwave balloon (the ratio of the balloon to the reference diameter is 1:1, the larger size is selected when the reference diameter is inserted between the two models, and the maximum balloon size, namely 4 mm, is selected when the vessel diameter is >4.0 mm); (2) Prepare the shockwave balloon using standard techniques: fill the syringe with 5cc saline/contrast agent (50:50), and connect the syringe to the filling port of the catheter seat. Vacuum at least three times to make the liquid replace the air in the catheter; (3) the shockwave balloon is placed at the target lesion site; (4) inflate the balloon to 4 atm for 10 pulses in the first cycle; (5) increase the inflation pressure to the nominal pressure, that is, 6 atm; (6) deflate the balloon and ensure that there are no remaining bubbles; (7) wait at least 10 seconds for distal vascular perfusion; (8) repeat steps 4–7, repositioning the balloon if necessary (Figure 3).^1^

### 3.4 Clinical evidence

In June 2017, the U.S. Food and Drug Administration and the European Community approved IVL for the treatment of peripheral arterial calcification. In 2021, IVL was approved for use in coronary calcification.^1^ Four prospective, multicenter, and single‐arm clinical studies (Table 1), namely Disrupt CAD I [NCT02650128], Disrupt CAD II [NCT03328949], Disrupt CAD III [NCT03595176], and Disrupt CAD IV [NCT04151628] have laid a solid foundation for proving the safety and efficacy of IVL.^13^, ^14^, ^15^, ^16^

**Table 1:** Basic data of Disrupt CAD I–IV studies.

	Disrupt CAD I	Disrupt CAD II	Disrupt CAD III	Disrupt CAD IV
Time	2015.12–2016.12	2018.5–2019.3	2019.1–2020.3	2019.11–2020.4
Site	7 hospitals	15 hospitals	47 hospitals	8 hospitals
	(5 countries)	(9 countries)	(4 countries)	(1 country)
Number of patients	60	120	384	64
Age	72 (66–79)	72.1 ± 9.8	71.2 ± 8.6	75.0 ± 8.0
Male	48	94	294	48
LM	1	1	6	1
LAD	28	75	217	48
LCX	8	14	49	4
RCA	23	30	112	11
Lesion length, mm	18 (14, 25)	19.5 ± 9.8	26.1 ± 11.7^a^	27.5 ± 10.4
Reference vessel diameter, mm	3 (2.6, 3.2)	3.04 ± 0.53	3.03 ± 0.47^a^	2.9 ± 0.4
Stenosis of diameter, %	73 (59, 77)	60.0 ± 12.0	65.1 ± 10.8^a^	65.8 ± 10.9
Severe calcification	60	113	384	64
Number of IVL pulses	72 (40, 120)	70.7 ± 43.4	68.8 ± 31.9	104 ± 56
Operation success rate, %	95	94.2	NA	NA
Stent	60	120	381	NA
Acute luminal gain, mm	1.7 (1.3, 2.1)	1.63 ± 0.49	1.41 ± 0.48	1.67 ± 0.37
Incidence of MACE at 30 days, %	5 (3)	7.6 (9)	7.8 (30)	6.3 (4)
Residual diameter stenosis <50%, %	100	100	100^a^	100
Residual diameter stenosis <30%, %	92	100	99.5^a^	100

Abbreviations: IVL, intravascular lithotripsy; LAD, left anterior descending branch; LM, left main coronary artery; LCX, left circumflex artery; MACE, major adverse cardiac events; NA, not available; RCA, right coronary artery.

^a^ The total number included in the analysis was 381.

Disrupt CAD I study^13^ included 60 patients with severe calcification lesions, and the primary endpoint was success of operation (residual diameter stenosis <50% after stent implantation, no evidence of major adverse cardiac events [MACE] during hospitalization, namely cardiac death, myocardial infarction diagnosed by muscle brain isoenzyme of creatine kinase, or target vessel revascularization [TVR]). The primary safety endpoint was no MACE events within 30 days. The acute luminal gain after stent expansion was 1.7 mm. Three patients developed non‐Q‐wave MI, and the incidence of MACE was 5% within 30 days. 95.0% of patients met the primary efficacy endpoint. Two patients subsequently died of cardiac causes, which was not associated with operation, and the incidence of MACE rose to 8.5% at 6 months.

The Disrupt CAD II study,^14^ which included 120 patients, was designed to evaluate the safety and efficacy of the coronary IVL system before stent implantation. The primary endpoint was the incidence of MACE during hospitalization. Acute gain after stent expansion was 1.63 mm, and seven patients emerged non‐Q‐wave MI during hospitalization and 10 patients occurred MACE events (one Q‐wave MI, seven non‐Q‐wave MI, and one TVR). 94.2% of patients met the primary efficacy endpoint, and the 30‐day MACE incidence was 7.6%. In 47 patients who underwent OCT examination before IVL and before and after stent implantation, calcified fractures were found in 78.7% of coronary calcification plaques. And more than half of the calcified lesions had multiple fractures.

In the Disrupt CAD III study,^15^ 384 patients were followed up. The primary safety endpoint was no MACE event at 30 days after operation, and the primary effective endpoint was success of operation (defined as successful stent delivery with residual stenosis <50% checked by the core laboratory and no MACE event occurring in the hospital). Twenty‐nine hospitalized MACE events (one cardiac death, four Q‐wave MI, 22 non‐Q‐wave MI, and two target vessel revascularization) and 37 MACE events (two cardiac death, six Q‐wave MI, 23 non‐Q‐wave MI, and six TVR) were recorded. 92.2% of patients met the primary efficacy endpoint. The 30‐day incidence of MACE was 7.8%. In approximate 100 patients examined by OCT, the rate of area stenosis decreased significantly after IVL and further decreased after stent placement (*p* < .001). Calcified fractures were found in 67.4% of coronary calcification plaques, and multiple fractures were found in nearly half of the calcified lesions. One year follow‐up showed that the incidence of MACE was 13.8% (1.1% cardiac death, 10.5% MI, and 6.0% TVR) and the failure rate of target lesions was 11.9%, both of which were mainly due to non‐Q‐wave MI.

The Disrupt CAD IV study^16^ included 64 patients in Japan, with the same endpoint and MACE definition as the Disrupt CAD III study.^15^ Acute gain after stent expansion was 1.67 mm, and four patients developed non‐Q‐wave MI during hospitalization and nonhospitalization. 93.8% of patients met the primary efficacy endpoint, and the 30‐day MACE incidence was 6.3%. OCT results from 71 patients showed a significant reduction in the rate of area stenosis after IVL, with calcification fractures found in 53.5% of coronary calcification plaques. Nevertheless, multiple calcification fractures occurred in about one‐third of the study. The inconsistent efficacy of IVL in the treatment of calcified lesions may be related to the differences in the number of cases and regional calcification composition. Slow blood flow or no reflow was never observed in Disrupt CAD series studies.^13^, ^14^, ^15^, ^16^ But in Disrupt CAD III, severe dissection occurred in two cases, and perforation and acute occlusion were one case, respectively.^15^

In 2021, Liang conducted a comparative analysis of four single‐arm studies, summarizing the characteristics of the Disrupt CAD serial studies, and attempted to explore the differences of IVL and RA. Therefore, the ROTAXUS (NCT00380809) and PREPARE‐CALC (NCT02502851) studies were included in the analysis to evaluate the applicability and safety of IVL or RA. Since IVL and RA cannot be directly compared, it is only concluded the following results: (1) The procedural success rate of RA was high. (2) The incidence of target lesion revascularization and MACE was low. There was no difference between patients treated with RA or without RA.^17^

In the same year, Kereiakes performed a statistical reanalysis of clinical data from Disrupt CAD's I–IV series of studies. Between December 2015 and April 2020, a total of 628 patients enrolled at 72 sites in 12 countries were included. Due to the loss of follow‐up of two patients, 626 patients were eventually included. The purpose of this study was to explore the incidence of the primary safety endpoint (no MACE events), the primary efficacy endpoint (procedural success, defined as success in the absence of in‐hospital MACE and the core laboratory confirmed residual stenosis rate ≤30% after stent implantation), and secondary outcomes (severe CAG complications within 30 days, failure of target lesions, cardiac death, and stent thrombosis) after IVL at 30 days of follow‐up. The results of primary endpoints showed the incidence of MACE at 30 days was 7.3%, with 92.4% of patients meeting the primary efficacy and 92.7% of patients meeting the primary safety endpoints. The results of secondary endpoints displayed that target lesion failure, stent thrombosis, and cardiac death were 7.2%, 0.8%, and 0.5%, respectively. The incidence of post‐IVL and final severe CAG complications was 2.1% and 0.3%. The MACE‐free rate within 30 days was higher in patients with lesion length <25 mm than in patients with lesion length ≥25 mm. Moreover, the MACE‐free rate within 30 days and the success rate of operation were lower in patients with bifurcation lesions. The aggregated OCT results suggested that coronary artery lesions with calcification >25% on a single cross‐section might have several fractures after IVL. With the increase of calcification severity, the lesion location would be more prone to multiple cracks, and this change was independent of the depth of calcification lesions.^18^ A gender‐based subgroup study found there were no significant gender differences in safety and efficacy of IVL,^10^ and Kereiakes reached similar conclusions.^18^

IVL only involves the most basic interventional technology in the coronary drug and stent delivery system, namely balloon catheter, with low learning curve and greatly reduces training time.^18^ Although the IVL system is labeled as 6 F compatible, it can be used with a 5 F unsheathed guide catheter.^1^ At present, IVL has been used to treat ST‐segment elevation MI, left main artery diseases, chronic complete occlusion diseases, nodular calcification diseases, and eccentric calcification diseases.^10^ In one study, IVL was performed on six patients with intrastent restenosis and one patient with poor stent dilation. The mean follow‐up time was 200 days, and none of the seven patients experienced operation‐related complications or adverse cardiovascular events. The result confirmed that IVL could be used in patients with intrastent restenosis, further expanding the using range of IVL.^19^ It is worth mentioning that the effectiveness of IVL in multilayer stents has not been proven.^1^

### 3.5 Precautions and coping strategies

According to the Chinese Expert Consensus on Diagnosis and Treatment of Coronary Artery Calcification (2021 edition), contraindications of IVL mainly include the following five aspects: (1) failure of guide wires or IVL balloons to pass through the lesion; (2) bridge vascular lesions after coronary artery bypass grafting; (3) thrombotic lesions; (4) single coronary artery supplies blood flow; (5) CAG indicates the presence of dissection at the lesion site.^6^ In addition, about 13% of shockwave balloon ruptures during clinical use.^20^ When the degree of lesion stenosis or vessel distortion is significant, the rupture of balloon may lead to coronary artery dissection.^21^, ^22^, ^23^ Therefore, the application of IVL should be avoided in similar lesions. In addition to the above situations, there are also the following precautions in the process of applying IVL.

First, improper balloons may affect the efficacy of IVL. Although the ratio of the balloon to the reference diameter is recommended to be 1:1, the two values often do not match exactly in practice. Therefore, when the reference diameter of the target vessel is inserted between the two models, the larger size of the shockwave balloon is selected. And when the vessel diameter is >4.0 mm, the maximum size (4 mm) can be selected.^10^ When the target vessel diameter is more than 4 mm or there are severe eccentric plaques, the shockwave balloon is limited to fully attach to the vascular wall, and the therapeutic effect is reduced. The size of crossing profile of the IVL system are relatively large. In addition, in the case of severe lumen stenosis, the semi‐compliant balloon is required for pre‐expansion to promote the positioning and release of the shockwave balloon.^24^

Second, the following points should be paid attention to during operation: (1) The pressure of the shockwave balloon should not exceed the rated burst pressure. (2) The target lesion has at least 20 pulses. When a single IVL catheter is delivered to the maximum number of pulses (80 pulses, i.e., eight cycles), the catheter should be abandoned and >80 pulses should be avoided at the same lesion location.^10^ (3) If the calcification lesion is longer than 12 mm, the shockwave balloon can be repositioned in the case of sufficient dilation, with a minimum overlap of 2 mm recommended to prevent inadequate treatment.^1^ (4) Do not advance or withdraw the catheter unless the balloon is fully deflated under vacuum. If resistance is encountered, identifying the source of the resistance before proceeding will be necessary.

Third, the application of IVL may induce arrhythmia. The acoustic pressure waves produced by IVL are transmitted at a speed of 1 Hz. They can produce a rapidly decaying mechanical energy of 8–10 μJ without the presence of electrical stimulation in the surrounding tissues. When acoustic pressure waves activate stretch‐activated ion channels in the cardiac conduction system and depolarize myocardial tissue, the mechanical‐electrical coupling process of the heart is completed.^11^ Previous cases reported that IVL induced one case of atrial flutter, one case of atrial fibrillation, three cases of ventricular tachycardia, and one case of ventricular fibrillation.^25^, ^26^, ^27^, ^28^, ^29^, ^30^ Hill indicated that male, heart rate <60 beats/min, and total number of IVL pulses are independent predictors of arrhythmia.^15^ Sustained ventricular capture can be induced when ion channels are activated and the intrinsic heart rate <60 beats/min or RR interval >1 second. In clinical settings, 10 seconds with IVL may induce single atrial capture, single ventricular capture or >1 ventricular capture in patients with heart rate of 60 beats/min. Groups with heart rates <65 beats/min experienced IVL‐induced capture 16 times higher than those with heart rates ≥65 beats/min.^31^ During the testing of the newly implanted implantable cardioverter defibrillator, the electrical energy to induce ventricular tachycardia or ventricular fibrillation was 0.6–2.0 J. Kereiakes considered that the likelihood of inducing ventricular arrhythmias was very low.^11^ There were three cases of ventricular tachycardia^27^, ^28^, ^29^ and one case that ventricular ectopic pacing (induced by IVL pulse waves) fell in T‐wave of the previous cardiac cycle to generate ventricular fibrillation.^30^ The possibility about IVL‐induced ventricular arrhythmias could not be completely ruled out. Neleman thought that the occurrence of severe arrhythmias might be due to the special physique of the patients, and no cases about persistent ventricular arrhythmias by IVL have been found.^10^ For people with pacemakers and defibrillators, it is recommended to re‐evaluate pacemaker function after applying IVL.^1^ Therefore, a special electrocardiogram monitoring system should be configured during the use of IVL. If malignant arrhythmias occur, measures should be taken to maintain hemodynamic stability.

Finally, 49 interventional operations with IVL were performed in The People's Hospital of Liaoning Province of China in 2023. There was one quarter with long calcification lesions. One patient had paroxysmal ventricular tachycardia during the use of the shockwave balloon, and then spontaneously turned into sinus rhythm. Two patients had chest tightness with evidence of transient myocardial ischemia during the interventional operation, namely ST segment depression, but the processes were not terminated. Follow‐up after discharge confirmed the success of interventional therapy. Previous literature did not find relevant cases that IVL can induce myocardial ischemia, and these clinical cases further confirmed the importance of applying IVL‐dedicated electrocardiogram monitoring system.

## 4 LIMITATIONS OF TRADITIONAL TREATMENT METHODS

In the process of treating coronary calcification lesions, the efficacy of conventional coronary calcification modification techniques has been limited in varying degrees (Table 2). Inadequacy of noncompliant balloon dilation in target lesion may result in dissection or perforation due to pressure.^32^ The super high‐pressure balloon has limited crossability and may require the assistance of a guide extension catheter.^33^ There is a high risk of entanglement during the application of a Lacrosse nonslip element balloon.^6^ The use of cutting and scoring balloons in severe calcification lesions and a lesion that standard predilated balloons cannot pass are limited.^4^, ^8^ ELCA has limited efficacy in severe calcification lesions and is not routinely recommended. The incidence of vascular perforation and stent restenosis is about 3%, and the use of normal saline in coronary arteries may reduce the risk. The method of treatment is limited to when the guide wire of mechanical rotary grinding or microcatheter cannot pass through the lesion.^34^ Coronary RA or orbital atherectomy may not have sufficient radial effect on calcification lesions due to the variable movement of the guide wire of mechanical rotary grinding.^32^

**Table 2:** Advantages and disadvantages of methods for treating coronary calcification.

Methods	Advantages	Disadvantages
Noncompliant balloon	1. High‐pressure inflation with minimal increase in balloon diameter	1. Expansion of noncalcified quadrant masking under‐expanded or asymmetric stent expansion 2. Risk of intimal dissection and perforation
Super high‐pressure (OPN) balloon	1. Low entry‐profile balloon to easily pass and safely dilate the lesion 2. Low rate of major adverse cardiac events	1. Ddifficult to deliver for two layers design 2. Risk of dissection, stent stripping, longitudinal stent deformation, and perforation 3. Difficult to recross or reuse after inflation
Cutting balloon	1. Improvement of vessel compliance by creating discrete incisions in plaque 2. Reduced elastic recoil to prevent uncontrolled dissection 3. High rate of procedural success	1. Relatively little clinical evidence 2. High risk of procedural complications and perforation
Scoring balloon	1. Small crossing profile, good crossability, and an alternative method of cutting the balloon 2. Low risk of dissection and balloon slippage by special structure 3. Good efficacy intrastent restenosis	1. Relatively little clinical evidence
Lacrosse nonslip element balloon	1. Useful for mild to moderate calcification 2. Useful for preprocessing of mechanical rotary grinding	1. Risk of entangling with the guide wire and stent mesh 2. Risk of entangling nylon with rotating
Rotational atherectomy	1. High rotational speeds to facilitate easier lesion crossing and reduce the risk of burr entrapment 2. Useful for long segment of disease	1. Risk of a variety of peri‐procedural complications 2. Risk of rotawire fracture, perforation, and entrapment in severely tortuous vessels 3. Do not use or perform with significant caution in lesions with previous stents
Orbital atherectomy	1. Bidirectional ablation to reduce the risk of entrapment 2. Rapid setup with one single burr size 3. Force of the orbital spin for continuous blood flow to reduce thermal injury and coronary no‐reflow	1. Risk of localized wall injury
Excimer laser coronary atherectomy	1. Useful for mild to moderate calcification 2. An important auxiliary way of mechanical rotary grinding 3. Low risk of peri‐procedural complications	1. Relatively little clinical evidence
Intravascular lithotripsy	1. Excellent outcomes with low risk of peri‐procedural complications 2. Easy to operate and wide range of applications	1. Efficacy limited by crossing profile, diameter of the balloon, and plaque 2. Risk of dissection and inducing arrhythmia or ST segment changes

*Note*: A comparison of advantages and disadvantages about traditional calcification treatment techniques and intravascular lithotripsy.^4^, ^6^, ^8^, ^32^, ^33^, ^34^, ^35^

Unlike the traditional balloon‐based vessel preparation of high‐static barometric pressure, IVL generates acoustic mechanical energy with a low‐pressure inflated balloon, reducing the possibility of vascular injury. The IVL acoustic pressure wave is evenly distributed on the inflatable balloon and not affected by the location of the guide wire. IVL is effective in situ of calcification lesions, and decreases the risk of microthrombi breaking off and blocking distal vessels.^13^ The prospective, dual‐arm, multicenter, and noninferior ROTA.shock study^36^ analyzed the modification effect of IVL and RA on severe calcification lesions. The examination results of OCT before and after RA or IVL in 21 patients showed that RA produced greater acute lumen gain (RA: 0.46 ± 0.16 mm^2^ vs. IVL: 0.17 ± 0.14 mm^2^, *p* = .03). Even so, IVL resulted in more calcified plaque fractures (3.23 ± 0.49 vs. 1.67 ± 0.52, *p* < .001) and longer (IVL: 1.67 ± 0.43 mm vs. RA: 0.57 ± 0.55 mm, *p* = .01) fractures. A case of proximal calcification in the left circumflex artery (extending from the ostium of left circumflex artery to the left main stem) was reported with IVL (three cycles). OCT showed calcification cracked well and there was no extension of hematoma. After a stent placement in the left main stem (covering the proximal lesion and hematoma), CAG indicated favorable results.^37^ The ISAR‐CALC 2 trial (ClinicalTrials.gov: NCT05072730) is designed to demonstrate the advantages of using a super high‐pressure balloon for lesion preparation before stent implantation compared to IVL in severe coronary calcification. And the conclusion of the trial will be eagerly awaited.

## 5 A NEW ALGORITHM FOR CORONARY CALCIFICATION

At present, there are so few clinical trials comparing IVL with traditional treatment ways that normative guidelines for the treatment of calcified lesions including IVL have not been published. Based on the existing imaging examination and treatment methods of coronary calcification, we proposed a new treatment algorithm (Graphical abstract). When the imaging of CAG finds severe calcification lesions, it first tries to see if a balloon can pass the lesion. If the balloon passes, predilation with balloon can be applied, followed by further evaluation with IVUS or OCT. When the score of IVUS or OCT is lower than the threshold, the noncompliant balloon, cutting balloon, scoring balloon, super high‐pressure balloon, and Lacrosse nonslip element balloon will be used. Contrarily, to determine whether there is a viable deep calcification with IVL treatment or calcification nodules treated with mechanical rotary grinding, and in other cases both methods are acceptable. If the equipment of balloon, IVUS, or OCT cannot pass through the lesion, a microcatheter can be used. If the microcatheter can pass, it will be sent into a guide wire of mechanical rotary grinding; otherwise, the microcatheter will be abandoned and the guide wire of mechanical rotary grinding can be fed directly into the target vessel. To further determine whether the guide wire of mechanical rotary grinding can pass the lesion, if it can pass and it will be utilized. Conversely, ELCA can be applied. After the initial selection of treatment, IVUS or OCT is applied to assess whether cracks or lumen gains are present at the lesion. If the vascular compliance is improved to some extent, stent implantation will be performed. If the improvement is not significant, stent placement can be performed after IVL treatment. After stent implantation, the operation is completed when the final result is acceptable. If the result is not satisfactory, dilation by noncompliant balloon or super high‐pressure balloon and IVL can be performed after IVUS or OCT evaluation.

## 6 CONCLUSION

Any technology is constantly updated to achieve the best practical value, and IVL is no exception. Reducing the crossing profile of the balloon of IVL can optimize its crossability. Increasing the length and diameter of a single balloon of IVL or increasing the total pulse number of IVL can further reduce the time and cost of operation on the basis of expanding the application range. The existing evidence shows that IVL has great application prospects and potential application value in valvular heart diseases accompanied by calcification. Perhaps the emergence of IVL will set off a revolution in the treatment of coronary artery calcification.

## CONFLICTS OF INTEREST STATEMENT

The authors declare no conflict of interest.

## Supporting information

Sup 1 The calcification score of IVUS and OCT. Adapted from Ata [4] and Natthapon [8] et al. IVUS, intravascular ultrasound, OCT, optical coherence tomography.

## Data Availability

Data sharing not applicable to this article as no datasets were generated or analysed during the current study.
