# Supplementary figures and images for: Intravascular lithotripsy: A novel option for severe calcification of coronary artery
Source: Clin Cardiol. 2023 Nov 9;47(2):e24186. doi: 10.1002/clc.24186 (PMC10826240; doi:10.1002/clc.24186)

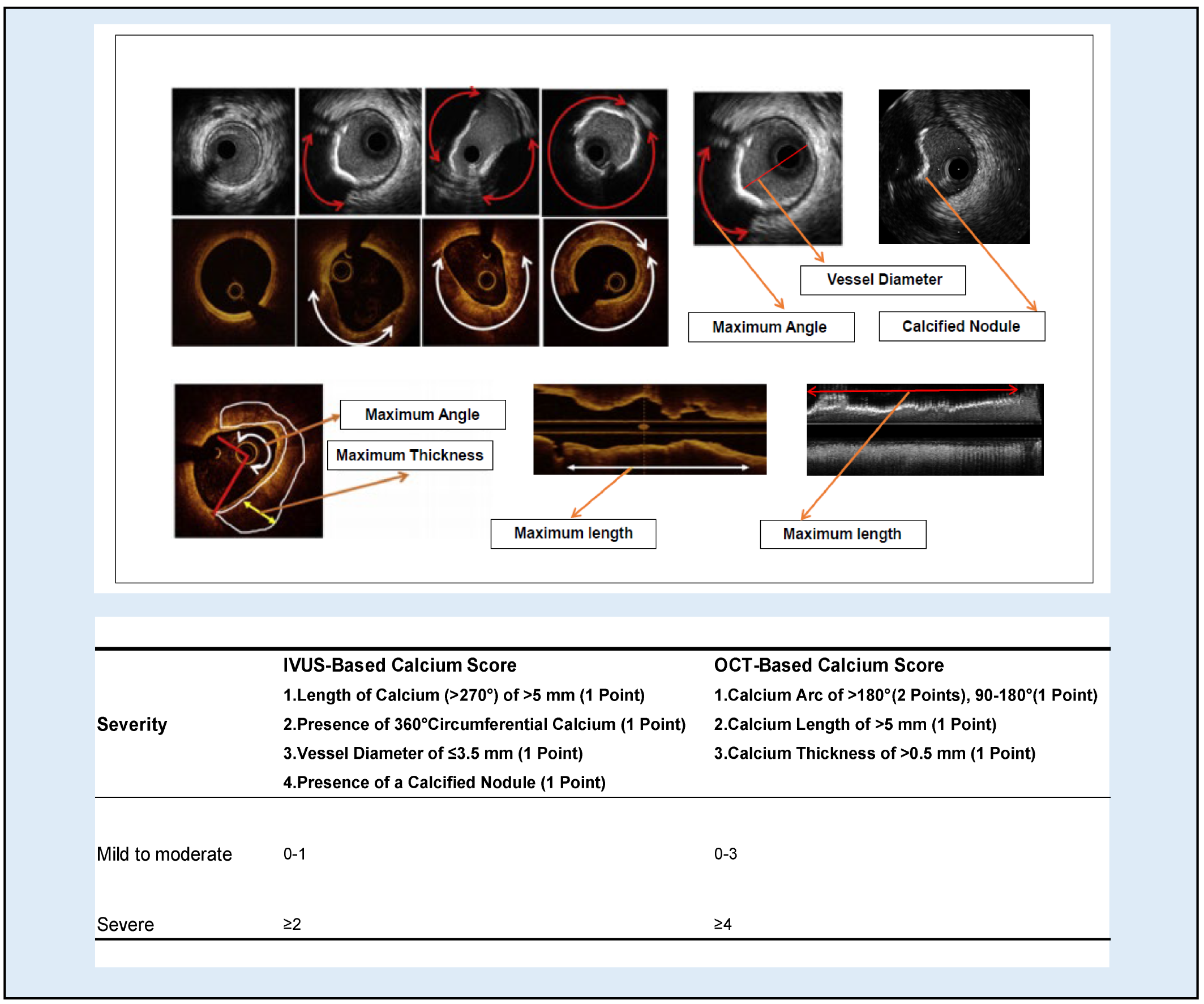

Supplement: Supplementary file 1 — Sup 1 The calcification score of IVUS and OCT. Adapted from Ata [4] and Natthapon [8] et al. IVUS, intravascular ultrasound, OCT, optical coherence tomography. [file CLC-47-e24186-s001.tif]
